# Macrophages in Collateral Arteriogenesis

**DOI:** 10.3389/fphys.2012.00353

**Published:** 2012-09-24

**Authors:** Erik Fung, Armin Helisch

**Affiliations:** ^1^Department of Medicine, Heart and Vascular Center, Dartmouth-Hitchcock Medical CenterLebanon, NH, USA

**Keywords:** monocytes, macrophages, collateral artery, vascular, arteriogenesis, growth, remodeling, angiogenesis

## Abstract

Arteriosclerotic vascular disease is the most common cause of death and a major cause of disability in the developed world. Adverse outcomes of arteriosclerotic vascular disease are related to consequences of tissue ischemia and necrosis affecting the heart, brain, limbs, and other organs. Collateral artery growth or arteriogenesis occurs naturally and can help restore perfusion to ischemic tissues. Understanding the mechanisms of collateral artery growth may provide therapeutic options for patients with ischemic vascular disease. In this review, we examine the evidence for a role of monocytes and macrophages in collateral arteriogenesis.

## Introduction

Arteriosclerotic disease is the major cause of death, and a major cause of morbidity and healthcare expenditure in the developed world. The clinical consequences of arteriosclerotic diseases are primarily related to the acute and chronic effects of tissue ischemia and necrosis affecting particularly the heart, brain, and limbs. Revascularization strategies have been employed with the goal of restoring blood flow to the ischemic tissue. Approaches to restoring blood flow through occluded or highly narrowed vessels include thrombolytic therapy, percutaneous interventional procedures (e.g., balloon angioplasty, stenting), and surgery (e.g., carotid endarterectomy, coronary artery bypass grafting).

In situations of acute ischemia presenting as acute myocardial infarction, stroke or acute limb ischemia, emergent restoration of blood flow by pharmacological therapy (thrombolytic therapy) or percutaneous interventional procedures is essential for limiting tissue necrosis. However, the risk/benefit ratio of revascularization procedures often becomes less clear in situations of chronic ischemia. Furthermore, the above approaches may not always be feasible for patients with severe diffuse occlusive arterial disease and poor target vessels for grafting. The costs and potential short- and long-term complications of surgical and percutaneous interventional procedures are not always offset by the variable extent of the benefits. The risks associated with current revascularization approaches include acute thrombotic occlusion or gradual restenosis of stents, and occlusion of bypass grafts. While coronary artery bypass grafting using certain arterial conduits (e.g., internal mammary artery) have relatively high patency rates of 85–90% after 10 years, venous conduits (e.g., saphenous vein grafts) are patent only 55–70% of the time after 10 years and 50–60% after 15 or more years (Fitzgibbon et al., 1996; Goldman et al., 2004; Tatoulis et al., 2004; Sabik et al., 2005).

Humans, like other organisms with vascular systems, have the capacity to grow vessels during early development as well as in adulthood. Functional studies on vascular growth began in 1785 with the Scottish anatomist, physiologist, and surgeon, Sir John Hunter (1728–1793), who documented his findings on the occlusion of the external carotid artery of a buck, and its physiologic consequences. He observed that the animal’s ipsilateral antler became cool to touch and its arterial pulse became impalpable, as its blood supply was dependent on the ligated artery. However, when the animal was reexamined a week later, the temperature and arterial pulsations of the antler had normalized. On further examination, Hunter found that small branches of the artery above and below the ligature had enlarged, and through their anastomoses the blood supply was restored (Murley, 1984). Well over two centuries later, this compensatory physiologic process, particularly at the cellular and molecular level, remains largely unclear. In this article, we review the different major mechanisms of natural vascularization and focus on the evidence for monocytes and macrophages in collateral artery growth.

### Vascular growth processes in developing and adult organisms

The vascular system is the first functional organ system in vertebrates. Vasculogenesis is the *de novo* formation of a primary capillary plexus from mesenchymal-derived progenitor or stem cells (Risau et al., 1988; Jin and Patterson, 2009). This is followed by sprouting and non-sprouting angiogenesis, arterial and venous specification, recruitment of smooth muscle cells with further arterial growth, differentiation, outward remodeling and inward remodeling/pruning (Risau, 1997; Carmeliet, 2005).

Vascular growth processes continue after birth and are necessary for maintaining physiologic homeostasis including organ growth, wound healing, and the menstrual changes of the uterine mucosa. Angiogenesis plays a role in many pathological conditions, ranging from inflammatory diseases to tumorigenesis (Carmeliet, 2005; Cao et al., 2011).

### Vascular growth processes in patients with atherosclerotic vascular diseases and in animal models of arterial occlusions

Vascular growth occurs in situations of arterial occlusive disease. In regions of tissue ischemia and tissue necrosis, capillary angiogenesis and arteriolar growth have been observed (Helisch and Schaper, 2003; Nickerson et al., 2009a; Mac Gabhann and Peirce, 2010). Since the initial report by Asahara, Isner and colleagues, the possibility of postnatal vasculogenesis in regions of tissue ischemia and tissue necrosis generated much interest (Asahara et al., 1997; Kalka et al., 2000; Moldovan et al., 2000; Urbich et al., 2003; Tepper et al., 2005). However, the contribution of postnatal vasculogenesis to compensatory blood vessel growth in adult organisms has been refuted by a number of studies failing to find evidence for any significant degree of bone marrow-derived cells transdifferentiating into vascular wall cells in arterial occlusion, tissue ischemia, hypoxia, or growth factor stimulation (Beck et al., 2003; Ziegelhoeffer et al., 2004; O’Neill et al., 2005; Capoccia et al., 2006; Grunewald et al., 2006; You et al., 2006; Nickerson et al., 2009a,b).

**Figure 1:**
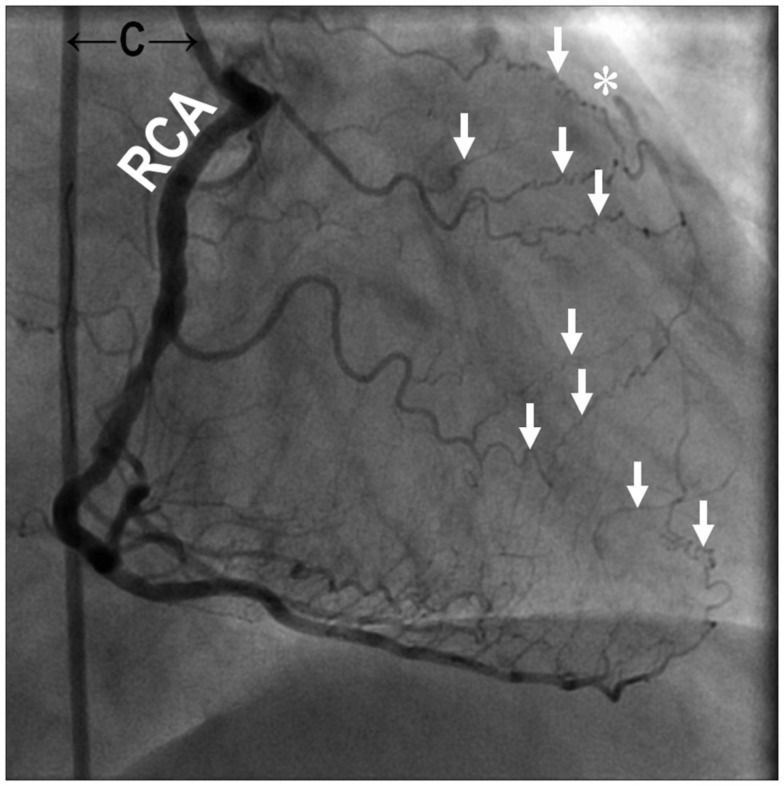
Coronary angiography of a 69-year-old male with chest pain (Canadian Cardiovascular Society Class IV) revealed occlusive coronary artery disease in the mid segment of the left anterior descending artery (*) bypassed naturally with collateral arterioles (↓) from the right coronary artery. The figure is taken from the cineangiogram in Supplementary Material during contrast injection into the right coronary artery. C, angiographic catheter; RCA, right coronary artery. Supplementary Material (Movie 1.MPG).

**Figure 2:**
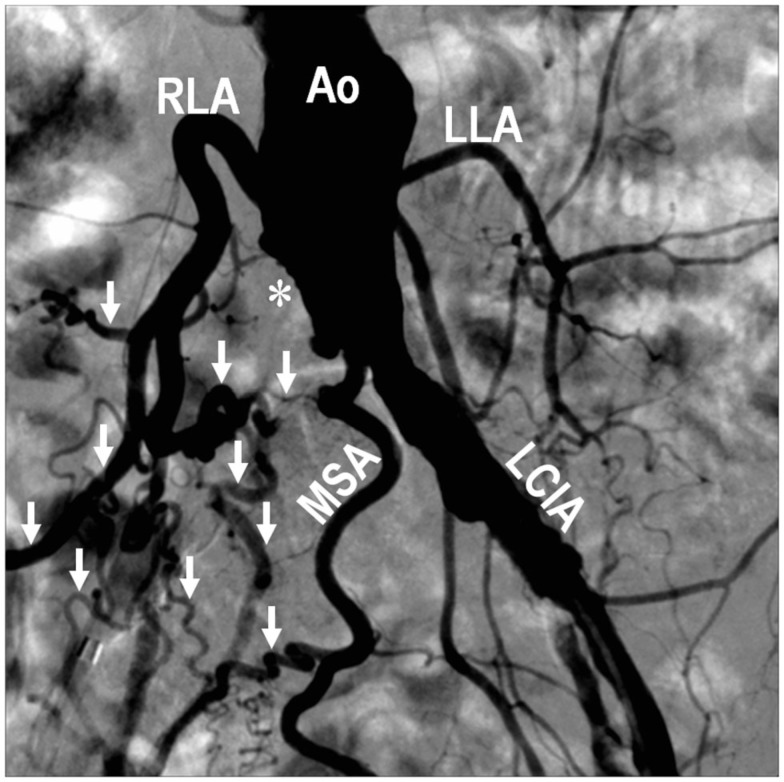
Digital subtraction angiography performed during abdominal aortography and peripheral arteriography on a 68-year-old female with lifestyle-limiting intermittent claudication showing an occluded right common iliac artery (*), and development of collateral vessels (↓) involving the middle sacral artery, right lumbar arteries, and other branches, to supply the right lower extremity. Compared with arteries on the left side, note the extensive outward remodeling on the right characterized by increased vessel diameter, tortuosity, and density as a consequence of the right-sided occlusion. Ao, abdominal aorta; LCIA, left common iliac artery; LLA, left lumbar artery; MSA, middle sacral artery; RLA, right lumbar artery.

The size, location, and connectedness of growing vessels are major determinants of their contribution to improving blood flow to ischemic tissue. For example, in a patient with proximal occlusion of a leg artery, severity of ischemia is worst in the distal portion of the limb, where capillary angiogenesis and arteriolar growth may occur. However, as the arterial occlusion is proximal, blood flow to the limb is primarily limited by the increased proximal arterial resistance and only arteries that functionally bridge or bypass the occlusion have the potential to completely compensate for the occluded artery (Unthank et al., 1995; Helisch and Schaper, 2003; Simons, 2005; Ziegler et al., 2010). Figure 1 and Movie 1 (Supplementary Material) are examples of a patient with complete arteriosclerotic occlusion of the mid left anterior descending artery that has been functionally bypassed by collaterals from branches of the right coronary artery (Figure 1). Figure 2 shows a complete arteriosclerotic occlusion of the right internal iliac artery in another patient in whom well-developed collateral arteries have formed. This process is distinct from other processes of vascular growth, and has been termed “collateral (artery) growth/remodeling,” “arteriogenesis,” or “collaterogenesis.”

### How do collateral arteries develop? *de novo* development or outward remodeling of preexisting vessels?

Direct vascular connections between arteries or arterioles have been demonstrated in many species and organs (Helisch and Schaper, 2003). These interarterial connections may be at the level of arteries, arterioles, and even capillaries (Helisch and Schaper, 2003; Mac Gabhann and Peirce, 2010). The circle of Willis at the base of the human brain and the arterial supply of the hands and feet of humans are examples of arterial networks where occlusion of one vessel may not result in any tissue ischemia, as blood flow through a parallel vessel may be adequate. Smaller, mostly arteriolar, preexisting interarterial connections are present in canine and human hearts and in the hind limbs of rodents (Herzog et al., 2002; Scholz et al., 2002; Helisch and Schaper, 2003; Wustmann et al., 2003; Helisch et al., 2006). After femoral artery ligation in different mouse strains, we observed the impact of differences in the innate collateral vasculature between BALB/c and C57BL/6 mice, and demonstrated, at least in the less ischemia-prone C57BL/6 strain, that collateral artery growth can be entirely based on the outward remodeling of innate collateral arterioles without any evidence for capillary angiogenesis in the region of collateral growth (Helisch et al., 2006). A recent elegant confocal microscopy study in spinotrapezius muscles revealed mouse strain-dependent differences in the skeletal muscle microvasculature, and demonstrated the ability of inter-arterially connecting capillaries to arterialize (Mac Gabhann and Peirce, 2010). Those findings lend further support to genetic (animal strain) differences being a source of inconsistency in the literature on experimentation on the innate collateral vasculature using different femoral artery ligations models.

While hypoxia is a potential stimulus for collateral arterial growth (Chilian et al., 2012), local tissue hypoxia is not essential for collateral arteriogenesis in the hind limb (Ito et al., 1997a; Deindl et al., 2001; Helisch et al., 2006) or the mesenteric circulation (Unthank et al., 1996). Increasing blood flow through innate collaterals by surgical manipulation (by creation of an arterio-venous shunt distal to the arterial ligation) appears to be one of the most powerful ways to increase their growth (Eitenmuller et al., 2006). This suggests that the mechanical forces of increased unidirectional blood flow in preexisting collateral arterioles after arterial occlusion are of major importance for their growth and maturation into collateral arteries, analogous to flow-induced remodeling known from other experimental models and clinical observations (Unthank et al., 1996; Helisch and Schaper, 2003; Schaper, 2009). The clinical example of peripheral vascular disease in Figure 2 illustrates how even large arteries enlarge as a consequence of the increased collateral blood flow.

Histologically, collateral artery remodeling is characterized by breakdown of the basement membrane, phenotypic modulation of vascular smooth muscle cells of the tunica media (from a contractile to a proliferative phenotype), cellular proliferation and apoptosis in all layers of the developing vessel, alteration in physical dimensions (the diameter and later the wall thickness of the vessel), and finally, reversion of the vascular smooth muscle cells to a contractile phenotype. The extent of growth in diameter and wall thickness tends to be much greater in dogs and rabbits than in rodents (Scholz et al., 2000, 2002; Cai et al., 2003; Helisch and Schaper, 2003; Schaper, 2009). Mice differ from non-rodent species in that a neointima is not observed during collateral arterial growth (Scholz et al., 2002).

While the remodeling of preexisting collateral vessels to collateral arteries has become an accepted mechanism for collateral artery growth, it remains unclear to what extent and under what conditions true *de novo* growth of collateral arterial vessels occurs, especially when the density of innate collaterals at the arteriolar or capillary level is low.

## Monocytes and Macrophages in Collateral Arteriogenesis

### Monocytes/macrophages accumulate in regions of growing collateral arteries

**Figure 3:**
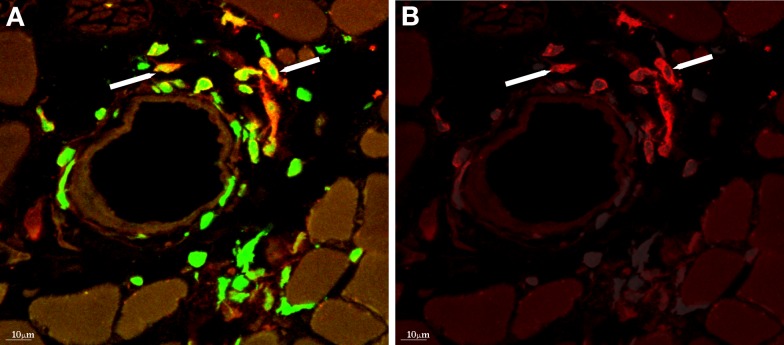
**(A)** Donor-derived cells (labeled with anti-GFP antibody, green) accumulating around collateral artery, after bone marrow transplantation from GFP expressing donor mice and femoral artery ligation. **(B)** Several of the bone marrow-derived cells express F4/80 (red), a marker for macrophages. With permission from (Ziegelhoeffer et al., 2004).

The concept that monocytes or macrophages play a role in collateral artery growth can be traced back to the pioneering histological studies of growing collateral vessels by Wolfgang and Jutta Schaper and their associates in the 1960s–1970s. After gradual coronary artery occlusion by ameroid constrictors in dogs, adherent monocytes were observed on the luminal side of the endothelium of growing collateral arteries, with evidence of diapedesis of these cells through the endothelium into the subintimal space (Schaper et al., 1976). Accumulation of monocytes/macrophages was observed around growing collateral arteries after femoral artery ligation in the hind limb of rabbits (Arras et al., 1998; Scholz et al., 2000), and mice (Scholz et al., 2002; Ziegelhoeffer et al., 2004). In these femoral artery ligation models, perivascular monocyte/macrophage accumulation peaked within the first 3 days, followed by a gradual decline at various times (Arras et al., 1998; Scholz et al., 2000; Khmelewski et al., 2004). Of note, other leukocyte subpopulations were also present around growing collateral vessels (Stabile et al., 2003, 2006; Ziegelhoeffer et al., 2004; Figure 3).

### Mechanisms of monocyte/macrophage recruitment and evidence for functional relevance in arteriogenesis

Recruitment of mononuclear phagocytes to tissues is a local and systemic process requiring chemotactic cytokines (chemokines) and adhesion molecules. The process is a continuum invoking bone marrow cell mobilization, monocyte emigration from bone marrow, chemotaxis, cell rolling along the endothelium, retention by adhesion, and transmigration through diapedesis (Luster et al., 2005; Serbina and Pamer, 2006; Rao et al., 2007).

Several studies have suggested that the number of circulating monocytes at or shortly after the time of arterial ligation correlated with collateral artery growth. These experiments involved the performance of arterial ligations in mice after depletion, or during the rebound phase, of circulating cells following administration of 5-fluorouracil (5-FU; Heil et al., 2002; Pipp et al., 2003), bisphosphonate-containing liposomes (Pipp et al., 2003), or reconstitution of monocytes around the time of surgery (Heil et al., 2002; Pipp et al., 2003; Cochain et al., 2010). These experimental approaches have broad physiologic effects, and thus, leave much room for data interpretation. For instance, cytostatic agents such as 5-fluorouracil do not selectively affect monocytes, and thus, the collateral growth promoting effects of the infused, crudely separated mononuclear cells would not be restricted to one specific cell subset, as claimed by a number of studies. Various types of mononuclear cells, have been referred to as “endothelial progenitor cells” or “circulating angiogenic cells” (Kalka et al., 2000; Rehman et al., 2003; Urbich et al., 2003), as well as adipose stromal cells (Rehman et al., 2004). The intramuscular administration of even platelets in a hind limb ischemia model of athymic nude rats reportedly augmented collateral arterial growth and associated with an accumulation of the injected cells around arterioles in the regions of arteriogenesis (Iba et al., 2002). Interestingly, a rat femoral artery ligation study using cyclophosphamide to deplete circulating cells did not find an effect on the number of accumulating macrophages around collateral arteries, or on arteriogenesis as assessed by bromodeoxyuridine (BrdUrd) uptake. Furthermore, the authors did not find any accumulation of fluorescently labeled blood cells around growing collateral vessels that had been injected intraarterially immediately after arterial ligation. The authors concluded that circulating monocytes did not accumulate around growing collateral arteries, and that the local proliferation of tissue-resident macrophages was of primary importance for collateral artery growth (Khmelewski et al., 2004). Concerns about that interpretation relate to the assessment of arteriogenesis using only BrdUrd uptake which may have lacked sensitivity for the detection of differences in collateral growth compared to methods of flow measurement. Furthermore, the continuous administration of intraarterial BrdUrd after arterial ligation could have labeled macrophage precursors before their emigration from the bone marrow. However, the possibility of *in situ* proliferation of the “M2” subpopulation of macrophages has recently been suggested by a study employing mouse models of pleural and peritoneal inflammation (Jenkins et al., 2011).

At least in cell culture, laminar flow can increase the expression of granulocyte-macrophage colony-stimulating factor (GM-CSF) in human umbilical vein endothelial and bovine aortic endothelial cells (Kosaki et al., 1998). This may be relevant for collateral arteriogenesis, as the mechanical forces of increased blood flow through preexisting collaterals after occlusion of a large artery may be the first alteration of the environment affecting endothelial cells of preexisting collateral vessels. Also, even transient shear stress-induced upregulation of monocyte chemoattractant protein (MCP-1; Shyy et al., 1994), intercellular adhesion molecule-1 (ICAM-1), and vascular cell adhesion molecule-1 (VCAM-1) have been reported; these factors are well-recognized in promoting monocyte recruitment and adhesion to the endothelium (Scholz et al., 2000; Rao et al., 2007). The observed expression of MCP-1, VEGF, and ephrinB2 on the endothelium of growing collateral vessels may be of importance for the recruitment of monocytes and macrophages *in vivo* (Scholz et al., 2000; Ziegelhoeffer et al., 2004; Korff et al., 2008).

Granulocyte-macrophage colony-stimulating factor promotes recruitment of monocytes/macrophages and arteriogenesis in animal models of hind limb (Buschmann et al., 2001) and cerebral ischemia (Buschmann et al., 2003; Schneeloch et al., 2004; Todo et al., 2008; Sugiyama et al., 2011). Subcutaneous GM-CSF was demonstrated to promote coronary collateral growth in a study of 14 patients with chronic stable coronary artery disease (Zbinden et al., 2005). Unfortunately, two of the seven patients in the GM-CSF group experienced acute coronary events, which did not occur in any of the seven control patients (Zbinden et al., 2005). In a study of 40 patients with peripheral vascular disease and claudication, repeated subcutaneous GM-CSF administration over 14 days did not influence the outcome as assessed by walking distance and ankle-brachial indices (van Royen et al., 2005).

Osteopetrotic (*Csf1^op^/Csf1^op^*) mice are deficient in colony-stimulating factor (CSF)-1, a growth factor that regulates the survival, proliferation, and differentiation of mononuclear phagocytic cells (Marks and Lane, 1976; Dai et al., 2002); these animals reportedly have impaired collateral artery growth, a reduction in the number of circulating monocytes and lymphocytes, and an increased number of circulating granulocytes (Bergmann et al., 2006). Granulocyte colony-stimulating factor (G-CSF) promoted macrophage (or leukocyte) accumulation and arteriogenesis in animal models of arteriogenesis in the hind limb (Lee et al., 2005; Capoccia et al., 2006) and brain (Sugiyama et al., 2011). Subcutaneous administration of G-CSF improved coronary collateral growth in a trial of 54 patients with chronic stable coronary artery disease (Meier et al., 2009).

MCP-1 is a ligand specific for C–C chemokine receptor (CCR2) with demonstrated importance in monocyte/macrophage recruitment. In studies using rabbit and porcine hind limb models of femoral artery ligation, Wolfgang Schaper and colleagues demonstrated that direct intraarterial infusion of MCP-1 led to an increase in collateral artery development in association with increased monocyte/macrophage accumulation around the growing collateral arteries (Ito et al., 1997b; Hoefer et al., 2001; Heil et al., 2002; Voskuil et al., 2003). A study using a femoral artery excision model reported that skeletal muscle tissue levels of MCP-1 were highest on day 3, paralleling the regenerative response in ischemic muscle (Shireman et al., 2006). MCP-1-deficient (*Ccl2^–/–^*) mice exhibited delayed or reduced collateral artery development or perfusion recovery in several independent studies (Voskuil et al., 2004; Shireman et al., 2007; Cochain et al., 2010) and consistently had abnormal monocyte recruitment (Lu et al., 1998; Voskuil et al., 2004). The MCP-1-induced increase of collateral growth can be abrogated by monoclonal antibody targeting ICAM-1 (Hoefer et al., 2004), highlighting the integral coupling of chemotaxis and leukocyte adhesion, and strengthening the concept of monocyte/macrophage-mediated arteriogenesis. Unfortunately, MCP-1 promotes not only arteriogenesis but also atherosclerosis (van Royen et al., 2003).

Following initial studies on MCP-1, focus was later shifted to its receptor, CCR2. CCR2 and CX_3_CR1 (fractalkine receptor) are probably the most well-studied chemokine receptors in monocytes/macrophages to date (Ancuta et al., 2003; Charo and Peters, 2003; Geissmann et al., 2003; Serbina and Pamer, 2006; Swirski et al., 2007; Tacke et al., 2007). Their differential surface expression levels can aid in classifying at least two major monocyte subsets defined by their physical properties (e.g., cell size, cytoplasmic granularity) and divergent physiologic functions (Geissmann et al., 2003, 2010; Gordon and Taylor, 2005; Fung et al., 2010; Butcher and Galkina, 2012). CCR2 and CX_3_CR1 play critical roles during infection, in maladapted inflammatory disorders, and in establishing homeostasis (Boring et al., 1998; Izikson et al., 2000; Serbina and Pamer, 2006; Auffray et al., 2007; Swirski et al., 2007; Tacke et al., 2007; Swirski, 2011). Promiscuity of CCR2 has raised challenges in the interpretation of studies using CCR2-deficient animals due to potential compensatory changes and overlapping properties of chemokines and adhesion mechanisms (Olson and Ley, 2002; Charo and Ransohoff, 2006; Shireman et al., 2007).

Studies using CCR2-deficient (*Ccr2^–/–^*) animals have reported variable results in restoration of perfusion following hind limb ischemia (Heil et al., 2004; Tang et al., 2004; Contreras-Shannon et al., 2007; Nickerson et al., 2009b). In a femoral artery ligation study a mildly decreased blood flow recovery with reduced perivascular and adventitial accumulation of macrophages in *Ccr2^–/–^* mice on C57BL/6 background was found; however a much more pronounced deficit in perfusion recovery and in gracilis muscle collateral arteriolar growth in *Ccr2^–/–^* mice on BALB/c background was observed (Heil et al., 2004). At least to some degree these background strain related differences in the effects of *Ccr2* deletion are probably related to differences in the innate collateral vasculature (Helisch et al., 2006). On the other hand, two other groups using a more severe ischemia surgical model observed unchanged perfusion recovery in *Ccr2^–/–^* compared to wild-type animals (Tang et al., 2004; Contreras-Shannon et al., 2007). In one study this was associated with decreased monocyte/macrophage recruitment only in the more ischemic calf muscle of *Ccr2^–/–^* mice, but not in the thigh, where collateral growth occurred (Tang et al., 2004), while the other one only assessed macrophages in the calf muscles; where decreased recruitment was observed. Interestingly, a more recent intravital microscopy study using mouse bone marrow chimeras and a dorsal skinfold window chamber model of injury/inflammation induced arteriolar remodeling reported significantly reduced accumulation of F4/80^+^ macrophages and abolition of arteriolar remodeling with *Ccr2* deficiency in bone marrow-derived cells (Nickerson et al., 2009b).

Mice with either MCP-1 or CXCR3 deficiency had similar decreases in their collateral arterial growth in the thighs, capillary density in the distal hind limbs, and in recovery of perfusion after femoral artery ligation, which was associated with a decrease in recruitment of macrophages and CD3^+^ T lymphocytes (Waeckel et al., 2005). In *Cxcr3-*deficient mice, vascular growth and perfusion recovery could be normalized by a single intravenous infusion of bone marrow-derived mononuclear cells from wild-type animals, but not from *Cxcr3*^–*/*–^ mice 5 h after femoral artery ligation (Waeckel et al., 2005), supporting the importance of CXCR3-expressing mononuclear cells in the very early stages of arteriogenesis. CXCR3 expression was also found to be essential for inward remodeling of the common carotid artery in response to decreased blood flow which is accompanied by adventitial macrophage accumulation (Zhou et al., 2010). CXCR3 deficiency reduced adventitial macrophage recruitment and abrogated inward remodeling as assessed by arterial diameter measurements (Zhou et al., 2010). Moreover, the authors found that impaired flow-mediated vascular remodeling in *Cxcr3^–/–^* animals could be restored by myeloid cells transferred from the wild-type counterpart, and that accumulation of perivascular macrophages was CXCR3-dependent. Extracellular matrix turnover mediated by macrophages was reportedly CXCR3-dependent (Zhou et al., 2010).

CX_3_CR1 and CX_3_CL1 (fractalkine) mediate the homeostatic functions of monocyte subsets, ranging from tissue and blood vessel patrolling (Auffray et al., 2007), monocyte recruitment in the spleen (Auffray et al., 2009) to their maladapted use in monocyte/macrophage accumulation in atherogenesis (Swirski et al., 2007; Tacke et al., 2007). The endothelium is a rich source of CX_3_CL1 that specifies migration and adhesion of monocytes and T cells (Bazan et al., 1997; Imai et al., 1997), and is a crucial gatekeeper in regulating leukocyte trafficking as well as a host of vascular and immune functions.

Local intramuscular injection of rat-specific CX_3_CL1 into the regions of collateral growth dose-dependently increased limb perfusion recovery after common femoral artery occlusion using an intraarterial coil approach, which avoids the problem of a local surgical wound (Ryu et al., 2008). While these studies implicated monocytes/macrophages in the process of neovascularization and arteriogenesis via the CX_3_CR1–CX_3_CL1 interaction, other circulating antigen-presenting cells including myeloid dendritic cells also express CX_3_CR1 and CD80 (B7-1), cell surface markers that have limited specificity in defining monocytes/macrophages. Furthermore, another study suggested that the CX_3_CR1–CX_3_CL1 interaction, which mediates recruitment of the Ly6C^lo^7/4^lo^ subset of monocytes, corresponding to the human CD16^+^CD14^lo^ “resident/patrolling” subset (Auffray et al., 2007), was unimportant for arteriogenesis compared to the CCL2/CCR2 pathway, which mediates recruitment of the “inflammatory” Ly6C^hi^7/4^hi^ subset-equivalent to the human CD14^hi^ CD16^−^ subpopulation (Cochain et al., 2010). Hind limb ischemia in this murine femoral artery ligation study was associated with a transient decrease of Ly6C^hi^7/4^hi^ cells in the bone marrow, supporting the hypothesis that these early accumulating cells are mobilized from the bone marrow. Interestingly, infusion of both monocyte subsets 6 h after femoral artery ligation resulted in an increase of vascular growth by angiographic score; however, only infusion of the Ly6C^hi^7/4^hi^ subset also resulted in an increased blood flow recovery (Cochain et al., 2010).

Recently, it was shown that the reticulon family member 4B (Nogo-B), which is known to be expressed by endothelial and vascular smooth muscle cells (Acevedo et al., 2004), is also expressed by macrophages and is important for some macrophage functions, including migration, spreading, and chemotaxis to MCP-1 and CSF. In a hind limb ischemia model, mice lacking Nogo-B had reduced collateral arteriogenesis and angiogenesis associated with a decrease in macrophage recruitment. Bone marrow reconstitution experiments showed that Nogo in myeloid cells plays a role in macrophage homing and blood flow recovery after limb ischemia (Yu et al., 2009).

### How may monocytes/macrophages promote collateral arteriogenesis?

Early studies on corticosteroids in modulating the development of collaterals suggested a role for inflammatory events in arteriogenesis (Borgers et al., 1973; Schaper et al., 1973). The first evidence for activated macrophages promoting angiogenesis was demonstrated by Polverini et al. (1977) in the avascular guinea pig cornea. While the potentiating effects of macrophages in neovascularization, especially tumor angiogenesis, are well-recognized (Mantovani et al., 2002; Pollard, 2004; Condeelis and Pollard, 2006), their potential effects in collateral artery growth have become a focus of study only since the late 1990s (Ito et al., 1997b; Arras et al., 1998).

While the ability of monocytes/macrophages to transdifferentiate into endothelial cells has been suggested based on *in vitro* studies (Fernandez Pujol et al., 2000; Schmeisser et al., 2001), evidence does not support this mechanism as relevant for arteriogenesis and even angiogenesis in ischemic conditions (Ziegelhoeffer et al., 2004; Capoccia et al., 2006; Nickerson et al., 2009a).

However, there is convincing evidence that monocytes and macrophages promote vascular growth in general, and arteriogenesis through paracrine effects. Monocytes and macrophages are capable of producing a large variety of growth factors, metalloproteinases, chemokines, vasoactive substances such as nitric oxide, all of which can facilitate arteriogenesis.

#### Growth factors

For a long time it has been known that activated macrophages can secrete substances which stimulate microvascular growth, as initially demonstrated in a guinea pig corneal angiogenesis model (Polverini et al., 1977). Monocytes/macrophages accumulating around collaterals express growth factors including VEGF-A (Ziegelhoeffer et al., 2004) and fibroblast growth factor (FGF)-2 (Arras et al., 1998; Ziegelhoeffer et al., 2004) that promote proliferation of endothelial and vascular smooth muscle cells (Sato et al., 2000; Schaper, 2009; Poling et al., 2011) which are essential processes for arteriogenesis. Growth factors also have chemotactic effects, as shown by the effect of VEGF-A on macrophages via VEGF receptor 1 (VEGF-R1). VEGF-A–VEGF-R1 signaling may be the mechanism by which placental growth factor (PGF), a selective VEGF-R1 agonist, augments macrophage recruitment to regions of collateral artery growth (Luttun et al., 2002; Pipp et al., 2003). Furthermore, there is compelling evidence that certain growth factors have direct tissue protective effects in situations of ischemia unrelated to vascular growth *per se* (House et al., 2003; Suzuki et al., 2005).

#### Metalloproteinases

MMPs are the primary proteolytic enzymes responsible for extracellular matrix remodeling. MMPs are present in and around growing collateral arteries (Cai et al., 2003) and are essential for collateral artery growth in a repetitive coronary occlusion model (Dodd et al., 2011), and in a mesenteric arterial ligation model (Haas et al., 2007). Monocytes and macrophages secrete MMPs in large quantities; however, the relative contribution of MMP secretion by macrophages to the increased MMP expression in and around growing collateral arteries has not been defined.

#### Nitric oxide and redox state modulation

Monocytes/macrophages express inducible nitric oxide synthase (iNOS). When induced, macrophages release abundant amounts of nitric oxide in their antimicrobial defense against invading pathogens, generating damaging concentrations of reactive oxygen intermediates and peroxynitrite (ONOO^–^) that are normally kept at low levels by superoxide dismutase, catalase, and peroxidase at rest (Wong and Goeddel, 1988; Schmidt and Walter, 1994). This may be relevant for arteriogenesis as well, since the importance of the redox state on arteriogenesis has been reported (Rocic et al., 2007).

The contribution of nitric oxide to arteriogenesis has previously been examined in experiments using NOS inhibitors (e.g., *N*-methyl-l-arginine-acetate or L-NNA), nitric oxide donors (e.g., *S*-nitroso-*N*-acetylpenicillamine or SNAP), *eNOS^–/–^* knockout and eNOS transgenic mice, and an exercise training animal model with acute arterial occlusion (Yang et al., 2002; Prior et al., 2003; Cai et al., 2004; Mees et al., 2007). eNOS contributes to NO-mediated vasodilation of peripheral collateral vessels, but its contribution to arteriogenesis *per se* is still controversial (Yang et al., 2002; Prior et al., 2003; Cai et al., 2004; Mees et al., 2007). Using *iNOS^–/–^* knockout mice, a study suggested that iNOS may play a significant role in arteriogenesis, as abrogation of arteriogenesis in *eNOS^–/–^* mice could only be achieved with addition of a relatively selective iNOS inhibitor, l-N6-(1-iminoethyl)lysine (l-NIL; Troidl et al., 2010). Moreover, the nitric oxide donor, diethylenetriamine (DETA) NONOate strongly promoted collateral arteriogenesis and activated perivascular monocytes that led the authors to postulate that shear stress on monocytes may explain the effects of iNOS in arteriogenesis (Troidl et al., 2010). Flow and shear stress have been shown to induce iNOS in endothelial, vascular smooth muscle cells, and mononuclear phagocytes/macrophages (Stuehr et al., 1990; Beasley et al., 1991; Schmidt and Walter, 1994).

#### Cytokines

TNF-α is expressed by monocytes/macrophages around growing collateral arteries (Arras et al., 1998). Mononuclear phagocytes, especially the differentiating CD14^+^CD16^+^ macrophage-like monocytes in transition from CD14^+^ inflammatory “classical” monocytes, and differentiated macrophages are major sources of TNF-α (Fingerle et al., 1993; Ziegler-Heitbrock et al., 1993; Belge et al., 2002; Kawanaka et al., 2002). TNF-α is a proinflammatory cytokine that has been reported to be important for arteriogenesis either related to its activation of TNF receptor 1 (Hoefer et al., 2002) or, in a more severely ischemic hind limb model, TNF receptor 2 (Luo et al., 2006). We found neither deficiency of TNF-α/β nor that of TNF receptor 1 or 2 to affect perfusion recovery after femoral artery ligation in mice (Helisch et al., 2003). Thus, the exact role of TNF-α in arteriogenesis, remains unclear, also in light of a recent study reporting M2-skewed macrophages as effectors of arteriogenesis (Takeda et al., 2011). However, the release of TNF-α is suppressed by IL-4 and IL-13, the Th2 cytokines that promote M2-skewing of macrophages (Stein et al., 1992; Gordon and Martinez, 2010).

#### PR39

A specific macrophage-derived antimicrobial peptide, PR39, delivered into the myocardium via adenoviral gene transfer was identified to improve collateral artery growth in a pig model of chronic myocardial ischemia (Post et al., 2006), and in a murine hind limb ischemia model (Tirziu et al., 2005). In a mouse hind limb ischemia study using the protein, we observed a strong tissue protective effect of PR39 and related peptides, however, without effect on blood flow recovery (Helisch et al., 2002), and potentially explainable by anti-apoptotic effects (Wu et al., 2004). The mechanism of PR39 is interesting, in that that it enhances hypoxia-inducible factor-1α (HIF-1α)-dependent gene expression by selectively inhibiting proteasome degradation of this transcription factor (Li et al., 2000; Post et al., 2006). Adenoviral PR39 treatment resulted in increased local VEGF, VEGFR-1, VEGFR-2, syndecan, and FGF receptors (FGFR)-1 levels. In addition, PR39 also stimulates expression of the FGFR-1 and syndecan-4, all of which may contribute to arteriogenesis and tissue resistance to ischemia (Post et al., 2006).

### What is the importance of monocyte/macrophage polarization?

M2 macrophages have been ascribed pro-angiogenic properties not observed in M1 cells in studies of tumor angiogenesis (Mantovani et al., 2009). A recent paper suggested that macrophage skewing also plays a role in arteriogenesis (Takeda et al., 2011). Haploinsufficiency of prolyl hydroxylase-2 (PHD2), an oxygen sensor involved in the ubiquitin mediated proteasomal degradation of hypoxia-inducible factor, resulted in an M2-like macrophage phenotype based on results from transcriptional profiling of *Phd2*^+/−^ murine peritoneal macrophages (Takeda et al., 2011). This study was quite surprising in that the tissue protective effects of *Phd2*^+/−^ monocytes/macrophages seemed at least in part related to their ability to increase the preexisting collateral vasculature before arterial ligation! This effect on the preexisting collateral vasculature was associated with an increased accumulation of tissue macrophages even at baseline. The skewing toward a pro-arteriogenic phenotype reportedly relied on the activation of the canonical NF-κB pathway and was associated with an increased production of SDF-1 and PDGF-B (Takeda et al., 2011).

## Conclusions

Collateral arteriogenesis is an important natural compensatory mechanism in situations of tissue ischemia related to occlusive arterial diseases. Understanding how collateral arteriogenesis can be augmented may lead to development of therapeutic approaches for patients with ischemic vascular diseases.

Recruitment of monocytes/macrophages to regions of collateral artery growth has been observed in various animal models. Initiation of the process requires mechanical forces related to the increase of blood flow through innate collateral vessels occurring after arterial occlusion. Although tissue ischemia may contribute as a stimulus, collateral growth has been clearly demonstrated to occur without local tissue ischemia. Published studies suggest that monocytes/macrophages mediate arteriogenesis via paracrine effects.

The extent of the effect of interventions designed to inhibit or augment monocyte/macrophage recruitment in arteriogenesis or arteriolar remodeling in animal models has varied widely from insignificant to large. The varying extent and severity of tissue ischemia and necrosis in different models may explain some of the outcome differences. In models with severe initial tissue ischemia, final recovery may not primarily depend on vascular growth processes, as these take time, however on the initial degree of ischemia and tissue loss. Furthermore, we are not aware of an animal model that provides a complete and selective elimination of monocytes and macrophages. Thus, in our opinion, the degree of monocyte/macrophage contribution to arteriogenesis remains uncertain. The details of macrophage recruitment to preexisting collateral vessels, and the potential importance of macrophage subsets and skewing in arteriogenesis will remain to be defined.

In humans, the role of monocytes/macrophages in collateral development remains unclear. The impaired formation of collaterals in human diabetics may be due to impaired chemotaxis of monocytes (e.g., to VEGF-A; Waltenberger et al., 2000; Tchaikovski et al., 2009). In a study of 16 patients with coronary artery disease, transcription profiling of monocytes showed differences between patients with well-developed collateral vessels compared to patients without angiographically visible collateral vessel.

The therapeutic efficacy of macrophage-related therapies remains questionable. Data from clinical trials of GM-CSF and G-CSF suggested an increase in collateral-dependent flow; however, the largest (*n* = 40 patient) study of patients with peripheral artery disease did not show a difference between the intervention and control groups. As discussed above, activating macrophages (and angiogenesis) can worsen atherosclerosis, as shown in animal studies (e.g., MCP-1), and can trigger acute ischemic events (as suggested in a clinical trial using GM-CSF).

A better understanding of myeloid cells and their subsets in collateral arterial remodeling, and their involvement in atherogenesis, atherosclerotic plaque stability, and tissue resistance to ischemia, will be essential for the investigational field of “therapeutic arteriogenesis.”

## Conflict of Interest Statement

The authors declare that the research was conducted in the absence of any commercial or financial relationships that could be construed as a potential conflict of interest.

## Supplementary Material

The Supplementary Material for this article can be found online at http://www.frontiersin.org/Vascular_Physiology/10.3389/fphys.2012.00353/abstract
